# Structural and Functional Modulation of Gut Microbiota by Jiangzhi Granules during the Amelioration of Nonalcoholic Fatty Liver Disease

**DOI:** 10.1155/2021/2234695

**Published:** 2021-12-20

**Authors:** Rui-rui Wang, Lin-fang Zhang, Lu-ping Chen, Jian-ying Wang, Lei Zhang, Yue-song Xu, Pei-lin Yang, Guang Ji, Bao-cheng Liu

**Affiliations:** ^1^Shanghai Innovation Center of TCM Health Service, Shanghai University of Traditional Chinese Medicine, No. 1200 Cailun Road, Shanghai 201203, China; ^2^Oxford Suzhou Centre for Advanced Research, Building A, 388 Ruo Shui Road, Suzhou Industrial Park, Jiangsu 215123, China; ^3^Institute of Digestive Diseases, Longhua Hospital, Shanghai University of Traditional Chinese Medicine, No. 725 South Wanping Road, Shanghai 200032, China

## Abstract

Recently, accumulating evidence revealed that nonalcoholic fatty liver disease (NAFLD) is highly associated with the dysbiosis of gut microbiota. Jiang Zhi Granule (JZG), which is composed of five widely used Chinese herbs, has shown hypolipidemic effect, while whether such effect is mediated by gut microbiota is still unclear. Here, we found that both low and high doses of JZG (LJZ and HJZ) could improve hepatic steatosis and function, as well as insulin resistance in NAFLD mice. 16S rRNA gene sequencing revealed that JZG treatment could reverse the dysbiosis of intestinal flora in NAFLD mice, exhibiting a dose-dependent effect. Notably, HJZ could significantly reduce the relative abundance of Desulfovibrionaceae, while increasing the relative abundance of such as S24_7 and Lachnospiraceae. PICRUSt analysis showed that HJZ could significantly alter the functional profile of gut microbiota, including the reduction of the lipopolysaccharide biosynthesis and sulfur metabolism pathway, which is verified by the decreased levels of fecal hydrogen sulfide (H_2_S) and serum lipopolysaccharide binding protein (LBP). In addition, hepatic mRNA sequencing further indicated that the HJZ group can regulate the peroxisome proliferator-activated receptor (PPAR) pathway and inflammatory signaling pathway, as validated by RT-PCR and Western blot. We also found that different doses of JZG may regulate lipid metabolism through differentiated pathways, as LJZ mainly through the promotion of hepatic lipid hydrolysis, while HJZ mainly through the improvement of hepatic lipid oxidation. Taken together, JZG could modulate gut dysbiosis with dose-effect, alleviate inflammation level, and regulate hepatic lipid metabolism, which may subsequently contribute to the improvement of NAFLD. Our study revealed the underlying mechanisms in the improvement of NAFLD by a Chinese herbal compound, providing future guidance for clinical usage.

## 1. Introduction

NAFLD is one of the most common chronic liver diseases worldwide, and its incidence is rising rapidly [1–4]. NAFLD encompasses a spectrum of liver diseases including isolated hepatic steatosis, nonalcoholic steatohepatitis (NASH), cirrhosis, and hepatocellular carcinoma [5, 6]. Although multiple potential therapies targeting the pathophysiological processes of NAFLD have been tested, few medications are clinically available owing to efficacy and safety issues [7–9]. Thus, novel and effective therapies for the treatment of NAFLD are still needed.

The gut microbiota is the largest microbial community in the human body, playing important roles in the host homeostasis [10]. Studies have found that obesity and NAFLD are strongly associated with specific changes of gut microbiota, such as lower microbial diversity and reduced Firmicutes-to-Bacteroidetes (F/B) ratio [11–13]. It has also been reported that the diet-induced intestinal mucosal inflammation and gut barrier disruption, which increase the likelihood of bacteria and bacterial product translocation, are closely associated with the progression of NAFLD [14]. Remarkably, when germ-free lean mice were transplanted with the cecal microbiota from obese mice or human, the recipient mice showed increased hepatic triglyceride accumulation [15–17], revealing the causal role of gut microbiota in the development of NAFLD. In addition, the severity of NAFLD is also associated with the shift of structure and metabolic function of gut microbiota [16]. The metabolites produced by gut microbiota have also been involved in the pathogenesis of NAFLD, including lipopolysaccharide (LPS), short-chain fatty acids (SCFA), bile acids, and tryptophan [16, 18–20]. Therefore, targeting at gut microbiota might be a promising therapeutic strategy for the prevention and treatment of NAFLD.

Many herbal remedies and their active components used in traditional Chinese medicine (TCM) have shown overt anti-obesity and anti-NAFLD effects, while gut microbiota has been implied as one of the important targets [21–27]. Jiangzhi granules (JZG) is a classic traditional Chinese compound medicine, which is developed in accordance to the theory of traditional Chinese medicine and has been widely used for patients with NAFLD for more than a decade. Previous studies have found that JZG is an effective herbal formula for the treatment of NAFLD. Phase II clinical trials have confirmed its safety and effectiveness in the treatment of NAFLD patients [28], while animal experiments have confirmed that JZG can improve insulin resistance and reduce lipid accumulation in the liver [29, 30]. However, there is still no clinical or experimental study that evaluates the effects of JZG on the gut microbial dysbiosis. Therefore, our experiment intends to study whether JZG can prevent NAFLD through intestinal flora by analyzing the structural and functional changes of intestinal flora and related disease phenotypes.

## 2. Materials and Methods

### 2.1. Preparation of JZG

JZG was purchased from Sichuan Neo-Green Pharmaceutical Technology Development Co., Ltd., composed of *Gynostemma pentaphyllum* (*Thunb.*) *Makino* (15 g), *Polygoni cuspidati* rhizome (15 g), *Folium nelumbinis* (6 g), *Artemisia capillaris Thunb* (9 g), and *Salviae miltiorrhizae* Radix (9 g). JZG was administered at the low (497 mg/kg/d, LJZ) and high (994 mg/kg/d, HJZ) doses in mice, according to the clinical equivalent dose [29]. In previous studies, we have stated the approach for the identification and quantification of the main components of JZG [31].

### 2.2. Animal Experiment

6 to 8-week-old male C57BL/6 mice were purchased from Shanghai Research Center of Southern Model Organisms (Shanghai, China) and maintained in specific pathogen-free (SPF) environments. All animal experiments in this study were approved by the Animal Ethics Committee of Shanghai University of Traditional Chinese Medicine and conducted under the “Guide for the Care and Use of Laboratory Animals” recommended by the US National Institutes of Health.

After one week of acclimatization, mice were randomly assigned to two groups fed different chows: (1) normal chow diet group (NCD, *n* = 10) and (2) high-fat diet (D12492; Research Diet) group (HFD, *n* = 26). After 16 weeks of HFD diet, the NAFLD mouse model was evaluated to be successfully established by dissection of two mice in each group. Then, the HFD group was randomly divided into three groups of 8 mice each: (1) HFD group, fed a HFD and administered by 0.5% sodium carboxymethyl cellulose intragastrically once daily; (2) LJZ group, fed a HFD and administered LJZ intragastrically once daily; and (3) HJZ group, fed a HFD and administered HJZ intragastrically once daily. After 8-week administration, all animals were sacrificed under anesthesia, and the blood, liver, and intestinal tissues were harvested following refined protocols.

### 2.3. Serum and Hepatic Biochemical Analyses

The levels of serum alanine aminotransferase (ALT) and aspartate aminotransferase (AST) were determined using Catalyst One Chemistry Analyzer. Total triglyceride (TG), total cholesterol (TC), free fatty acids (FFAs), superoxide dismutase (SOD), and malondialdehyde (MDA) in extracted hepatic samples were measured with corresponding assay kits (Nanjing Jiancheng Bioengineering Institute, Nanjing, Jiangsu, China). Serum insulin and LPS-binding protein (LBP) were determined using ELISA kits (Crystalchem, 90080; Abcam, ab269542, respectively) according to the manufacturer's instructions.

### 2.4. Pathological Observation of Liver Tissue

The liver tissues of mice were isolated and fixed in 10% formalin solution for 48 h. The tissues were dehydrated, cleared, waxed, embedded, sliced, and stained with hematoxylin and eosin (H&E). The presence of steatosis was further confirmed using Oil Red O staining of frozen liver sections. The morphological differences of the tissues were examined under a light microscope, and all quantitation was performed in Image-Pro Plus software 6.0.

### 2.5. Gut Microbiota Analysis

To determine the structure and function profile of gut microbial community, we used Illumina high-throughput sequencing to sequence the 16S rRNA gene V3-V4 region of the gut microbiota in mouse feces. Total bacterial DNA was extracted from mice fecal samples using the DNeasy PowerSoil Kit (QIAGEN, Inc., Netherlands). Amplicon library for double-ended (2 × 300 bp) sequencing was constructed using the forward primer 338F (5′-ACTCCTACGGGAGGCAGCA-3′) and the reverse primer 806R (5′GGACTACHVGGGTWTCTAAT-3′) on Illumina MiSeq platform. PCR amplicons were purified with Agencourt AMPure Beads (Beckman Coulter, Indianapolis, IN) and quantified using the PicoGreen dsDNA Assay Kit (Invitrogen, Carlsbad, CA, USA). After quantification, amplicons were pooled in equal amounts. The raw reads were quality-filtered and merged [32]. Paired-end reads were assembled using FLASH. After chimera detection, the remaining high-quality sequences were clustered into operational taxonomic units (OTUs) at 97% sequence identity by UCLUST. A representative sequence was selected from each OTU using default parameters. OTU taxonomic classification was conducted by BLAST searching the representative sequence set against the Greengenes database using the best hit. To minimize the difference of sequencing depth across samples, an averaged, rounded rarefied OTU table was generated by averaging 100 evenly resampled OTU subsets under the 90% of the minimum sequencing depth for further analysis. OTUs containing less than 0.001% of total sequences across all samples were filtered. Raw sequences generated in the present study were deposited to NCBI Sequence Read Archive under accession number SRP334314.

### 2.6. SCFA Analysis

Cecal SCFA concentration was measured by gas chromatography/mass spectrometry (GC/MS). Briefly, 50 mg of cecal content was homogenized with 0.8 mL ddH_2_O and then 0.01 mL of 50% H_2_SO_4_ and 1 mL of diethyl ether. After vortexing for 30 s, the mixture was centrifuged at 4°C and 10000 rpm for another 5 min. 200 *μ*L of the supernatant was acquired and then measured by GC on an Agilent7000B system (Agilent Technologies, CA, USA) equipped with flame ionization, thermal conductivity detectors, capillary columns, and GC ChemStation software. Acetate, butyrate, propionate, valerate, isobutyrate, and isovalerate were quantified using pure standards diluted in diethyl ether.

### 2.7. Hydrogen Sulfide Determination

The fecal supernatant was prepared according to the ratio of 1 mg feces compared to 2 *μ*L phosphate buffer (pH 7.2). The fecal supernatant was mixed thoroughly before centrifugation at 16000 rpm for 15 min at 4°C. The supernatant was collected and centrifuged again for 10 min. 100 *μ*L of the supernatant was acquired and then was homogenized with 100 *μ*L phosphate buffer solution and 10 *μ*L of Sulphide I reagent (Hach Chemical, Dusseldorf, Germany, 1816-20032). After standing for 5 s, 10 *μ*L of Sulfide II reagent (Hach Chemical, Dusseldorf, Germany, 1817-32) was added into the mixture. After standing another 5 min at room temperature, the absorbance of the mixture was determined at a wavelength of 665 nm by a Hach DR 5000 spectrophotometer (Hach Chemical, Dusseldorf, Germany) [33].

### 2.8. RNA Sequencing

Total RNA was extracted from the livers of the NCD, HFD, and HJZ groups (*n* = 3) which were randomly selected. An RNA Seq library was constructed using Illumina TruSeq RNA Sample Preparation Kit, according to the manufacturer's instructions. We then used the PCR amplification to enrich the library fragments as long as 300-400 bp. Then, the Agilent 2100 Bioanalyzer was used for quality inspection of the library. Then, next-generation sequencing technology (Next-Generation Sequencing, NGS) was used to perform paired-end sequencing of these libraries based on the Illumina HiSeq sequencing platform (Personalbio, Shanghai, China). Then, we use HTSeq to compare the Read Count value of each gene as the original expression of the gene and use FPKM to normalize the expression.

### 2.9. Reverse Transcription PCR

Total RNA extracted from the liver and colon tissues was reverse-transcribed to cDNA using a SuperScript™ first-strand synthesis system for reverse transcription PCR (RT-PCR) (Invitrogen, United States). RT-PCR was performed on a LightCycler96 (Hangzhou Bioer Technology, Share-Holding Co.) using iQ SYBR Green Supermix (BIO-RAD, United States). The relative expression of lipid synthesis-related genes (Scd1, PPAR*γ* [34]), lipid hydrolysis-related genes (Adrb3, Lipe, Pnpla2 [35]), lipid oxidation-related genes (Cpt2, Acox1, Ppargc1a [35], PPAR*α* [36]), lipid transport gene (Fabp5 [37]), intestinal permeability-related genes (Occludin, ZO-1, Muc5 [38]), and inflammation-related genes (CD14 [39], TLR2, TLR4, NLRC4, and MCP-1 [40]) were adjusted with Glyceraldehyde-3-phosphate dehydrogenase (GAPDH) as the housekeeping gene. Relative quantification was calculated using the 2^−∆∆Ct^ method. The primer sequences for RT-PCR can be found in Supplementary Table 1.

### 2.10. Western Blot Analysis of Hepatic Proteins

Mouse liver tissues were lysed in RIPA buffer with 1× protease and phosphatase inhibitor cocktail to extract total protein. The total protein concentration in the supernatant was determined using the BCA method. After samples were denatured at 100°C for 5 min, equal amounts of proteins were run on a 10% SDS-PAGE gel and then transferred onto a PVDF membrane. Membrane was blocked with 5% nonfat milk in 0.1% TBST for 2 h at room temperature and then incubated with a primary antibody overnight at 4°C. After extensive washing, the membranes were incubated with the appropriate HRP-conjugated secondary antibody at room temperature for 2 h. Finally, the blots were developed with an ECL reagent. The following primary antibodies were used: GAPDH (Monoclonal Antibody (2B8), YM3029, 1 : 5000), PPAR*α* (Polyclonal Antibody, ab215270, 1 : 500), Fabp5 (Polyclonal Antibody, YN2385,1 : 5000), and CD14 (Polyclonal Antibody, 17000-1-AP, 1 : 500).

### 2.11. Immunohistochemistry

The hepatic PPAR*α* and intestinal Occludin expression were evaluated using paraffin-embedded liver and ileum tissues, respectively. After deparaffinization, the slides were heated in an autoclave with sodium citrate for antigen repairing, followed by 1% hydrogen peroxide to abolish endogenous peroxidase activity, and blocked with 2% goat serum. Slides were then incubated with primary antibodies including PPAR*α* (Servicebio, GB11163, 1 : 20) and Occludin (Servicebio, GB111401, 1 : 500) at 4°C overnight. HRP-conjugated secondary antibodies (1 : 200) were incubated for 50 min at room temperature. After PBS washing, peroxidase substrate DAB (Dako, K5007) was used for color development.

### 2.12. Statistical Analysis

Data were presented as the means ± standard deviation. Graphs are depicted as violin plots to display the distribution of the data. Statistical differences in the biochemical index and the distance of the gut microbiota between groups were assessed by the Mann–Whitney *U* test; multiple group comparisons were made by the Kruskal-Wallis test or one-way ANOVA, using GraphPad Prism 8.4 software. *p* < 0.05 was considered statistically significant. Other statistical analyses were described in the corresponding figure legends.

## 3. Result

### 3.1. JZG Improved the Metabolic Phenotype of HFD Induced NAFLD Mice

**Figure 1:**
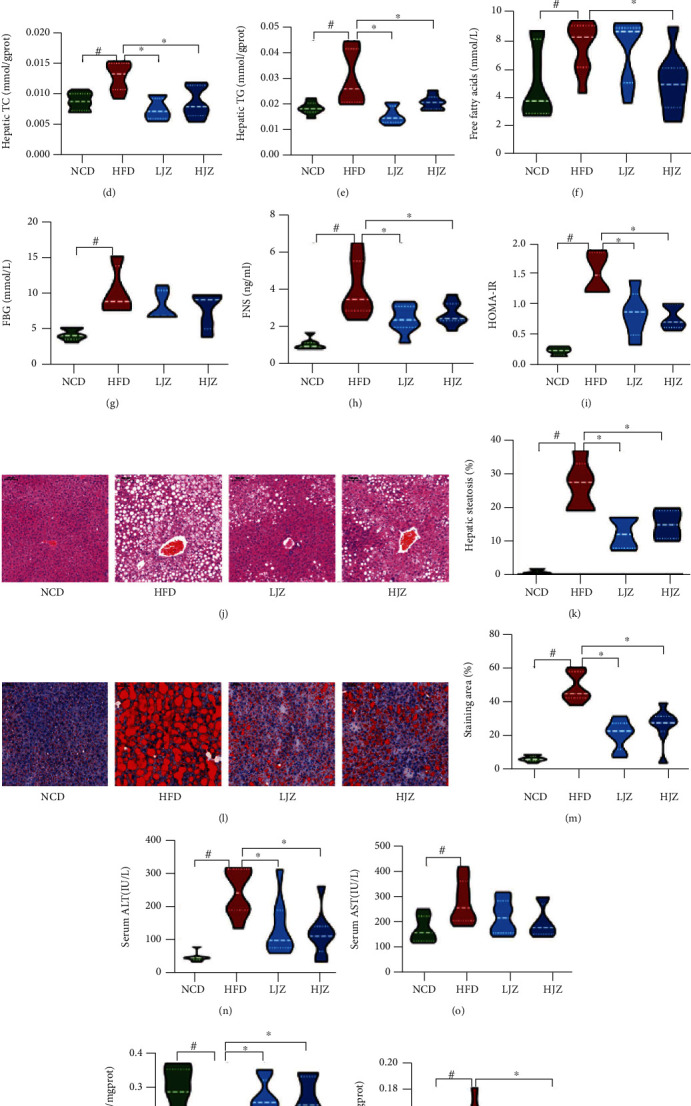
Effects of JZG on liver steatosis and function in HFD-fed mice: (a) body weight curve; (b) body weight gain rate; (c) food intake; (d) hepatic TC level; (e) hepatic TG level; (f) hepatic free fatty acids level; (g) fasting blood glucose; (h) fasting serum insulin; (i) HOMA-IR; (j) the HE-stained liver section; (k) bar graph of the volume density of liver steatosis; (l) oil red O-stained liver section; (m) the quantitative results of the oil red O staining; (n) serum ALT level; (o) serum AST level; (p) hepatic SOD activity; (q) hepatic MDA level. ^#^*p* < 0.05 compared with the NCD group. ^∗^*p* < 0.05 compared with the HFD group. Violin plots display medians with interquartile ranges.

After the establishment of NAFLD model by 16 weeks HFD, different doses of JZG were administered intragastrically for 8 weeks to investigate the therapeutic mechanism of JZG. As shown in Figure 1(a), the body weight in the HFD group was significantly higher compared to that in the NCD group, while the body weight in the HJZ group was significantly lower than that in the HFD group at the end of the experiment. The body weight gain rate in the LJZ and HJZ groups were significantly lower than those in the HFD group (Figure 1(b)), with no significant difference in dietary energy intake between groups (Figure 1(c)). These results indicated that JZG can reduce the body weight of mice under HFD conditions and the differences in mouse body weight were not caused by different dietary intake.

Furthermore, biochemical parameters in the serum and liver revealed that hepatic TC and TG levels were significantly increased in the HFD group compared to the NCD group and were significantly decreased in the LJZ and HJZ groups compared to the HFD group (Figures 1(d) and 1(e)). Hepatic FFA level was higher in the HFD group than in the NCD group and was significantly reduced in the HJZ group, too (Figure 1(f)). Serum insulin and HOMA-IR levels were also significantly decreased in the LJZ and HJZ groups compared to the HFD group (Figure 1(i)). Pathological examination of liver HE staining sections revealed extensive micro/macrovesicular steatosis and frequent incidence of hepatocyte ballooning in the HFD group, which were significantly ameliorated in livers of the LJZ and HJZ groups (Figures 1(j) and 1(k)). Oil-red O staining also showed that lipid deposition occurred in nearly 47.73% in the liver tissue of the HFD group, and JZG treatment significantly reduced HFD-induced liver lipid deposition (Figures 1(l) and 1(m)). Apart from that, compared with the HFD group, both the LJZ and HJZ groups could degrade the serum ALT level and improve the peroxidase activity in the liver to the extent of NCD group (Figures 1(n) and 1(p)), while the HJZ group but not LJZ group could decrease the hepatic level of MDA (Figure 1(q)). Our results showed that JZG could improve liver function and liver lipid deposition in a mouse fed a HFD.

### 3.2. JZG Reshaped the Gut Microbiota Structure of HFD-Induced NAFLD Mice

**Figure 2:**
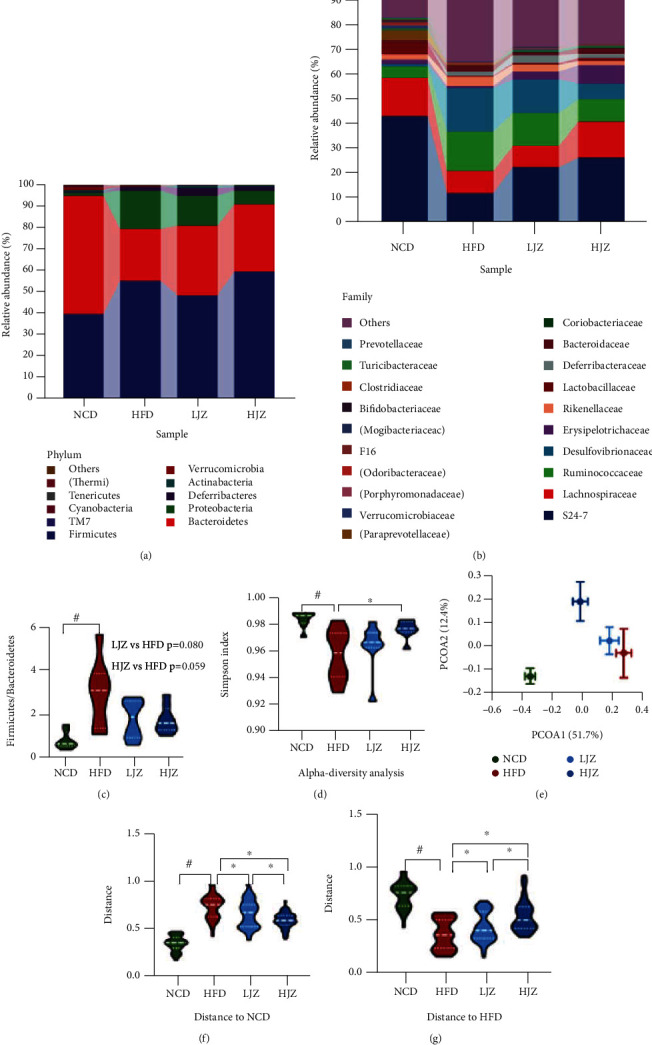
Effects of the JZG on structure of gut microbiota. (a) Bacterial composition at the phylum level; (b) bacterial composition at the family level; (c) F/B ratio; (d) alpha-diversity analysis with Simpson index; (e) PCoA analysis of four groups based on weighted UniFrac metrics, each spot represent for one group with mean ± SD; (f) distance compared to the NCD group through the Anosim algorithm; (g) distance compared to the HFD group through the Anosim algorithm. ^#^*p* < 0.05 compared with the NCD group. ^∗^*p* < 0.05 compared with the HFD group. Violin plots display medians with interquartile ranges.

In order to explore the potential involvement of the gut microbiota in mediating the improvement of NAFLD by JZG, we performed 16S rRNA gene sequencing of all the fecal samples at the end of the experiments. At phylum level, Firmicutes, Bacteroides, and Proteobacteria were the main components of the gut microbiota in our mouse model. The abundance of Proteobacteria, which consisted of many opportunistic pathogens, was significantly increased in the HFD group, while significantly decreased in both the LJZ and HJZ groups (Figure 2(a)). Meanwhile, HFD could significantly augment the F/B ratio while JZG showed the trend to decrease the F/B ratio (LJZ vs. HFD: *p* = 0.08; HJZ vs. HFD: *p* = 0.059) (Figure 2(c)). At family level (Figure 2(b)), JZG, especially the HJZ, showed the trend to reverse the change of bacterial composition induced by HFD, characterized by the higher abundance of S24_7 and Lachnospiraceae and lower abundance of Desulfovibrionaceae and Ruminococcaceae. Furthermore, the Simpson index reflected a decreased diversity in the HFD group compared to the NCD group (*p* < 0.05) and increased diversity in the HJZ group compared to the HFD group (*p* < 0.05) (Figure 2(d)). Beyond this, *β*-diversity based on weighted UniFrac distance indicated significant differences in the microbiome composition among groups (Figure 2(e)). The gut microbial community composition of the JZG group is significantly closer to that in the NCD group, while significantly distant to that in the HFD group, with a clear dose effect (Figures 2(f) and 2(g)).

**Figure 3:**
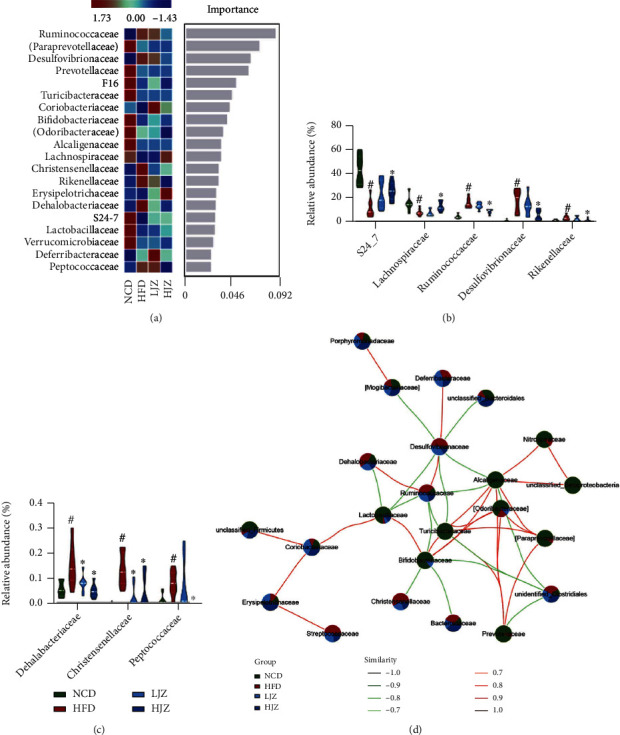
Differentiated bacteria between groups and network analysis. (a) Random forest analysis at the family level; (b) high and (c) low abundantly different families evaluated by the Wilcoxon rank-sum test; (d) network analysis of high abundant families with positive interactions in red and negative interactions in green. ^#^*p* < 0.05 compared with the NCD group. ^∗^*p* < 0.05 compared with the HFD group. Violin plots display medians with interquartile ranges.

To further identify the key bacteria, we performed differential analysis based on the random forest analysis and Wilcoxon rank-sum test. In all, 20 families were identified as the discriminative families among four groups through random forest analysis, and Ruminococcaceae, Paraprevotellaceae, and Desulfovibrionaceae were ranked as the highest discriminative families (Figure 3(a)). All these families were further tested by the Wilcoxon statistical test, and eight families showed significantly difference (Figures 3(b) and 3(c)). Compared with the HFD group, the HJZ group showed significant enrichment of S24_7 and Lachnospiraceae and significant reduction of Ruminococcaceae, Desulfovibrionaceae, Rikenellaceae, Dehalobacteriaceae, Christensenellaceae, and Peptococcaceae. Subsequently, we used the network analysis of high abundance families to study the interaction mode of microbial communities. The NCD group showed a cluster of closely correlated bacteria including Turicibacteraceae, Bifidobacteriaceae, Alcaligenaceae, and Lactobacillaceae, while JZG could recover partial of them although under HFD. The abundant bacteria such as Desulfovibrionaceae, Ruminococcaceae, and Dehalobacteriaceae were correlated while JZG could decrease the abundance of these bacterium (Figure 3(d)).

### 3.3. JZG Altered the Bacterial Functional Profile of HFD-Induced NAFLD Mice

**Figure 4:**
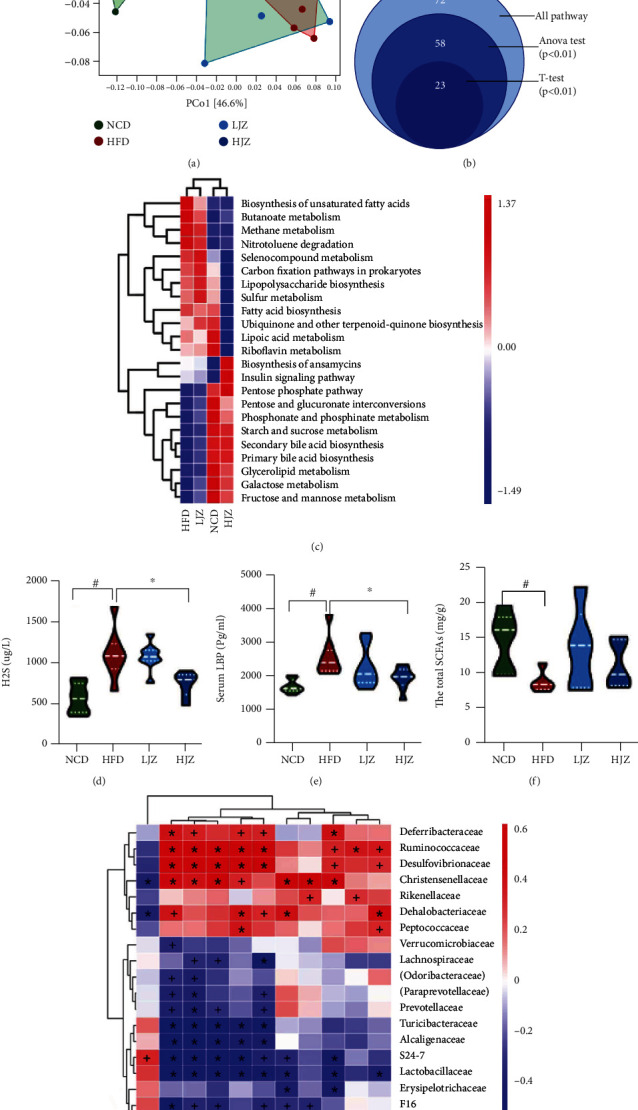
Effects of the JZG on bacterial function. (a) PCoA plot of the bacterial functions based on Bray-Curtis distances; (b) Venn diagrams of differentially KEGG metabolic pathways; (c) heatmap of different pathways between groups; (d) H_2_S content in feces; (e) plasmatic concentration of LBP; (f) fecal SCFAs in cecal contents. ^#^*p* < 0.05 compared with the NCD group. ^∗^*p* < 0.05 compared with the HFD group; (g) Spearman's correlations between gut microbial community at the family level and vital metabolic parameters linked to NAFLD; ^+^*p* < 0.05 and ^∗^*p* < 0.01. Violin plots display medians with interquartile ranges.

Changes in the structure and symbiotic relationship of gut microbiota also indicate functional changes. We then performed PICRUSt analysis to predict the function of the gut microbiota. The PCoA analysis of all the bacterial functions based on Bray-Curtis distances revealed that HFD could change the functional profile of the gut microbiota while the HJZ could alter the changes resembling to that in the NCD group (Figure 4(a)). 23 KEGG pathways showed significant difference through multiple statistic test (Figures 4(b) and 4(c)). The cluster analysis of the 23 KEGG pathways revealed that the HJZ group was clustered with the NCD group, similar with the result of PCoA. Specifically, we found that pathways of lipopolysaccharide biosynthesis, sulfur metabolism, and fatty acid biosynthesis were significantly lower in the NCD and HJZ groups, while pathways of primary and secondary bile acid biosynthesis and starch and sucrose metabolism were significantly higher in the NCD and HJZ groups (Figures 4(b) and 4(c)). These results indicated HJZ could recover the functional profile of gut microbiota to the NCD group.

Bacteria-associated metabolites were further detected to verify the results of PICRUSt. HJZ could significantly reduce the increase of fecal concentration of H_2_S and plasma concentration of LBP induced by HFD (NCD vs. HFD: *p* < 0.05, HJZ vs. HFD: *p* < 0.05, respectively) (Figures 4(d) and 4(e)). The total SCFA content was significantly lower in the HFD group compared to the NCD group (*p* < 0.05), while there was no difference between the HFD group and JZG group (Figure 4(f)). The correlations between the key bacteria and important metabolic parameters associated with NAFLD were presented in Spearman's correlation heatmap (Figure 4(g)). We found that bacteria enriched in HFD group such as Deferribacteraceae, Ruminococcaceae, and Desulfovibrionaceae were positively correlated with metabolic parameters associated with NAFLD (increased body weight; serum levels of ALT, AST, LBP and faecal level of H_2_S; and decreased level of SOD), while bacteria enriched in the NCD and JZG group such as S24_7, Lachnospiraceae, and Bifidobacteriaceae were negatively correlated with NAFLD-associated metabolic parameters.

### 3.4. Pathways and Genes Mediating the Therapeutic Effects of JZG Treatment

**Figure 5:**
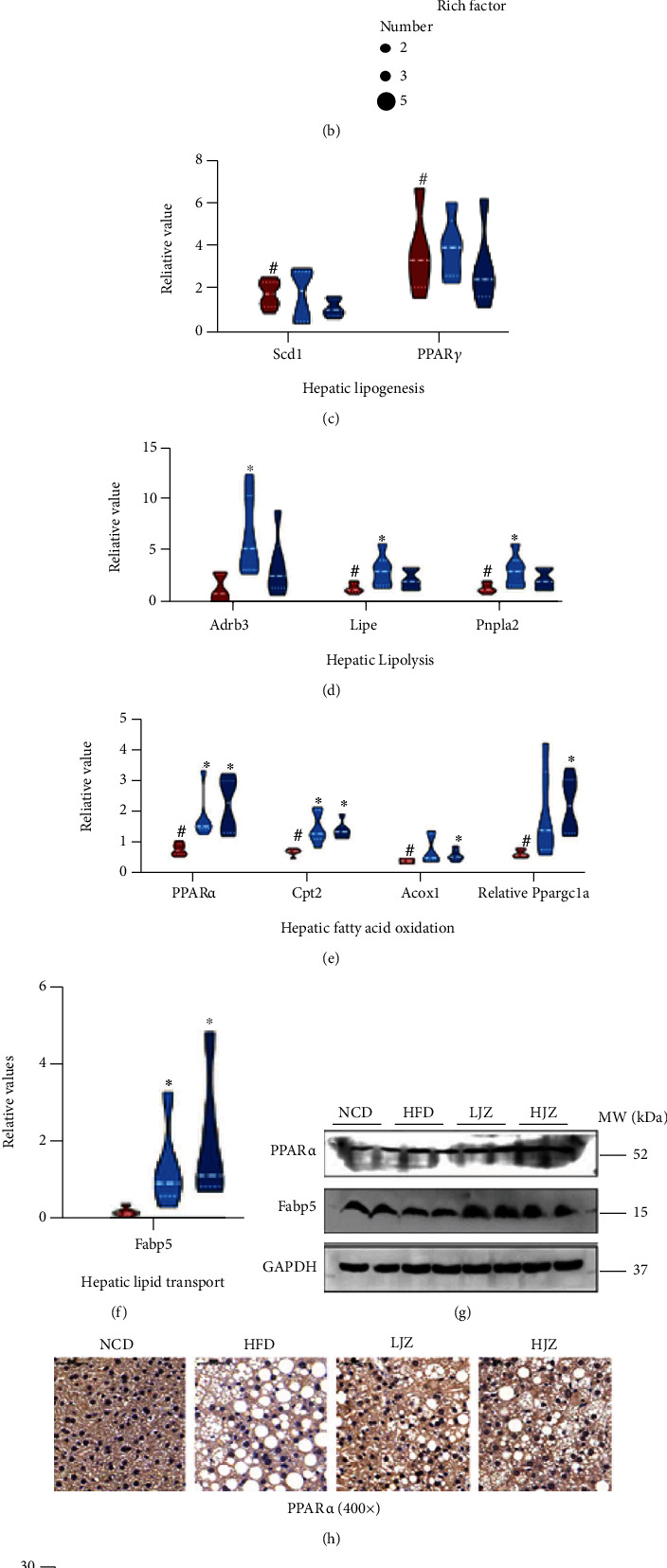
The potential mechanism of the JZG at the molecular level. (a) Enrichment analysis of KEGG metabolic pathway for the NCD group in comparison with that of the HFD group; (b) enrichment analysis of KEGG metabolic pathway for the HJZ group in comparison with that of the HFD group; compared with NCD group, the relative expression of genes encoding for lipogenesis (c), lipolysis (d), fatty acid oxidation (e), and lipid transport (f) in the liver; (g) Western blotting analysis of PPAR*α* and Fabp5 protein expression in the liver; (h) immunohistochemical staining for PPAR*α* protein expression in the liver (magnification, ×400); compared with NCD group, the relative expression of genes encoding for inflammation in the liver (i) and in the terminal ileum (j); (k) compared with the NCD group, the relative expression of genes encoding for intestinal permeability in the terminal ileum; (l) Western blotting analysis of CD14 protein expression in the liver; (m) immunohistochemical staining for Occludin protein expression in the terminal ileum (magnification, ×400). ^#^*p* < 0.05 compared with the NCD group. ^∗^*p* < 0.05 compared with the HFD group. Violin plots display medians with interquartile ranges.

We then performed hepatic mRNA sequencing to analyze the metabolic changes on transcriptome level during JZG treatment. We found that compared with the NCD group, the expression of 479 genes was significantly upregulated and that of 459 genes was significantly downregulated by HFD. Meanwhile, compared with the HFD group, the expression of 58 genes was significantly upregulated and that of 57 genes was significantly downregulated by the HJZ group (Supplementary Figure 1). According to the annotation information of the genome, KEGG function enrichment analysis of the NCD group and HFD group, the HFD group and HJZ group were conducted based on the genes differentially expressed between groups. Compared with the NCD group, the HFD group showed regulated pathways related to fatty acid degradation, PPAR, and primary bile acid metabolism etc. (Figure 5(a)), while the HJZ group showed regulated bile acid secretion, PPAR, and inflammatory signaling pathways compared to the HFD group (Figure 5(b)).

RT-PCR analysis was preformed to verify the transcriptome results on lipid metabolism and inflammatory signaling pathways. The expression of lipid synthesis-related genes (Scd1 and PPAR*γ*), lipid hydrolysis-related genes (Adrb3, Lipe, and Pnpla2), lipid oxidation-related genes (PPAR*α*, Cpt2, Acox1, and Ppargc1a), and lipid transport gene (Fabp5) in liver tissues was measured by RT-PCR. The results showed that, compared with the HFD group, the LJZ group significantly upregulated the expression of genes involved in lipid hydrolysis (Figure 5(d)) and the HJZ group significantly upregulated the expression of genes involved in lipid oxidation (Figure 5(e)); both the LJZ and HJZ treatments significantly increased the expression of the liver lipid transporter gene Fabp5 and decreased in some degree the expression of the hepatic lipid synthesis-related genes (Figures 5(c) and 5(f)). The higher expression of Fabp5 genes in the livers of the LJZ and HJZ groups was verified by Western blot, and the high expression of PPAR*α* genes in the livers of LJZ and HJZ groups was verified by Western blot and immunohistochemistry (Figures 5(g) and 5(h)). Meanwhile, the expression of inflammatory markers in the liver and intestine was further explored. Compared with the HFD group, both the LJZ and HJZ could significantly reduce the expression of CD14 gene in the liver and intestine, and the HJZ group could significantly reduce the expression of TLR2 gene in the liver. Moreover, both the LJZ and HJZ groups could reduce the expression of TLR4, NLRC4, and MCP-1 genes, although no statistic difference was detected (Figures 5(i) and 5(j)). Otherwise, we also found that compared with the HFD group, the LJZ could significantly increase the expression of Occludin, ZO-1, and Muc5 genes in the ileum, and HJZ could significantly increase the expression of Occludin (Figure 5(k)), indicating an improved intestinal permeability. Further, Western blot verified the results of decreased expression of CD14 gene in the liver of the LJZ and HJZ groups, and immunohistochemistry verified the results of higher expression of Occludin gene in the ileum of the LJZ and HJZ groups (Figures 5(l) and 5(m)).

## 4. Discussion

Many natural herbs have been reported to be effective in the treatment of metabolic diseases, while intestinal microbiota is highly supposed as one of the pivotal targets. In this study, we found that JZG, a Chinese herbal compound prescription, could improve hepatic steatosis and liver function in a NAFLD mouse model and further investigated the effects of JZG targeted at intestinal microbiota through multiomics approach. Our results showed that different doses of JZG could not only ameliorate gut dysbiosis in NAFLD mice with a dose-effect but also improve liver steatosis through differentiated regulatory pathways of lipid metabolism, as LJZ through the promotion of hepatic lipid hydrolysis, while HJZ through the improvement of hepatic lipid oxidation.

The present study showed that different doses of JZG have dose-dependent effect on intestinal microflora. Both doses of JZG could change the gut flora, while the structure and function of gut microbiota in the HJZ group were much more resembling to that in the NCD group, while diverging to that in the HFD group. Our finding is consistent with a previous intervention study that found higher doses of berberine and metformin played a better role in gut microbiota regulation [41]. Notably, we found Desulfovibrionaceae, typically reported as a notorious opportunistic pathogen which is closely related to obesity, T2DM, and colitis [42–45], is significantly decreased in the HJZ group. Desulfovibrionaceae is famous for its ability of sulfate reduction that could reduce sulfides into H_2_S, a toxic gas for colonic epithelial cells [46, 47]. Moreover, many species from Desulfovibrionaceae could release high content of LPS which shows high endotoxin activity and could metabolize SCFA which deprives colonic cells from their main energy source [48–52]. As functional validation, we also detected a decreased level of H_2_S and the LBP in the HJZ group [53]. Interestingly, a recent study has found that an acetic acid-producing bacteria *Desulfovibrio vulgaris* from Desulfovibrionaceae also showed anti-NAFLD effects [54], which highlight that we should emphasize on function evaluation instead of structural description of gut microbiota. Furthermore, we also found JZG could increase the abundance of beneficial bacteria such as S24_7 and Lachnospiraceae. S24_7 is a beneficial inhabitant in the gut and known as probiotics for its ability to degrade a variety of complex carbohydrates [55]. Lachnospiraceae is one of the most abundant butyrate-producing bacteria [56] which could regulate metabolism, immune response, and colonocyte growth [57]. We found that JZG could improve the cecal contents of SCFAs but no difference was detected between groups. Whether there is a difference content of SCFAs in the portal vein needs further investigation. So, JZG may play the role of prebiotic-like effect and could regulate the disturbance of intestinal flora caused by HFD through enriching beneficial bacteria and inhibiting harmful bacteria.

As a consequence of the improved dysbiosis of gut microbiota, we also found that JZG could improve the intestinal barrier function, the intestinal and hepatic inflammation levels, insulin resistance, and hepatic lipid accumulation. A previous study has reported that extraction from *Gynostemma pentaphyllum*, one vital ingredient of JZG, also could enrich beneficial intestinal bacteria and modulate inflammatory intestinal microenvironment [58]. Another study found that Andrographolide could exert antihyperglycemic effect through strengthening intestinal barrier function and increasing microbial composition of *Akkermansia muciniphila* [22]. Our results further indicated that JZG might reduce the HFD-induced inflammation by decreasing the CD14/TLR4 and TLR2 gene expression. CD14/TLR4 plays a vital role in the recognition of bacterial origin LPS [59] and induces cascade response of inflammation reaction, which leads to insulin resistance [60]. TLR2 expression is responsible for the recognition of various microbial components including Gram-positive bacteria-derived lipoproteins, peptidoglycan, and lipoteichoic acid, which could promote inflammatory response and metabolic adaption [61, 62]. JZG may regulate CD14/TLR4 and TLR2 signaling pathways, improving inflammation and insulin resistance targeted at gut microflora.

Finally, our study revealed the underlying mechanisms of the regulation of hepatic lipid metabolism by JZG with dose effect. Transcriptome results showed that the HFD group can inhibit fatty acid degradation and PPAR signaling pathways, while the HJZ group could reverse the obesogenic effects. Our results found that the HJZ group mainly increased the expression of genes related with fatty acid oxidation, while the LJZ group mainly increased the expression of genes related with lipolysis. A previous study of Flavokawain A also found a dose-effect of anti-inflammatory effects [63]. Another study of Tu-Teng-Cao (TTC) also observed that there was a dose-effect relationship between the TTC-M and TTC-L groups in the treatment of acute gouty arthritis [64]. This suggests that we should pay attention to the dosage of drugs during clinical treatment. The dose-effects on lipid metabolism might be induced by the discrepant gut microbiota shaped by different doses of JZG. Meanwhile, we also found JZG also increased the expression of hepatic PPAR*α* and Fabp5 on both RNA and protein levels. PPAR*α* is the master regulator of lipid metabolism in the liver by regulating the expression of genes involved in fatty acid uptake and intracellular trafficking, lipid deposition, and, mostly, *β*-oxidation [65]. Fabp5 represents a key transport protein of long-chain fatty acid and is involved in the regulation of biological reactions mediated by activation of PPAR [66, 67]. These results suggested that JZG regulates lipid metabolism via promoting lipid transport and metabolism rather than inhibiting lipid synthetic pathways, and there may be divergence regulation effects on the lipid metabolism pathway between different dose groups.

In conclusion, our study suggested that JZG could modulate bacterial composition and function with dose-effect, improve gut permeability, alleviate intestinal and hepatic inflammation level, and regulate liver lipid metabolism and then may subsequently ameliorate NAFLD. Our study shed light on further investigation of the clinical utility of Chinese herbal compound, which has been understudied in the treatment of NAFLD.

## Data Availability

16S rRNA data has been uploaded to NCBI: SRP334314 (https://dataview.ncbi.nlm.nih.gov/object/PRJNA757472?reviewer=nq9vec4iu9fh2lbndmfiigclj3).

## Supplementary Materials

Supplementary Materials Supplementary Figure 1: Venn diagrams showing the number of up- and downregulated genes among the NCD, HFD, and HJZ groups. Supplementary Table 1: the primer sequences for RT-PCR.
