# Supplementary material for: Structural and Functional Modulation of Gut Microbiota by Jiangzhi Granules during the Amelioration of Nonalcoholic Fatty Liver Disease
Source: Oxid Med Cell Longev. 2021 Dec 20;2021:2234695. doi: 10.1155/2021/2234695 (PMC8712166; doi:10.1155/2021/2234695)
Supplement: Supplementary Materials — Supplementary Figure 1: Venn diagrams showing the number of up- and downregulated genes among the NCD, HFD, and HJZ groups. Supplementary Table 1: the primer sequences for RT-PCR. [file 2234695.f1.pdf]

Supplementary materials

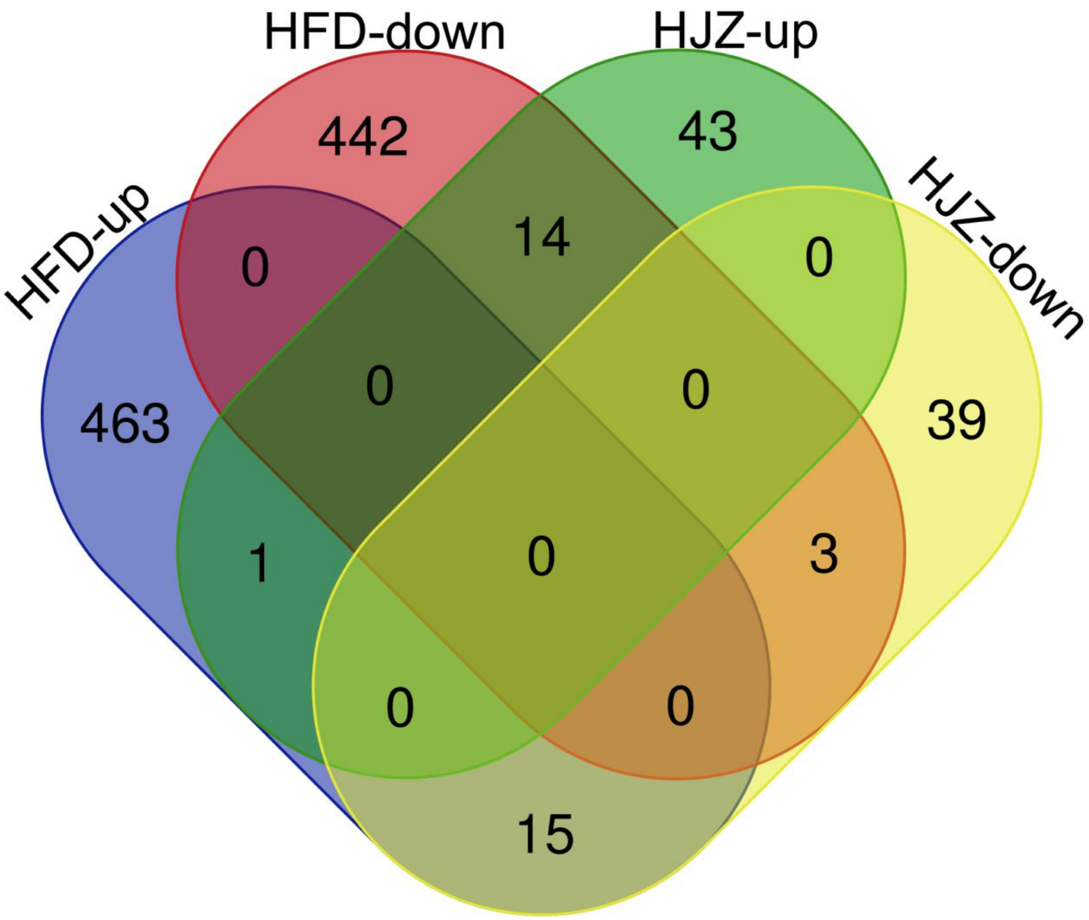

Supplementary figure 1. Venn diagrams showing the number of up- and down-regulated genes among NCD, HFD and HJZ group.

Supplementary Table 1 The primer sequences for RT-PCR

Gene Symbol	Forward Primer	Reverse Primer
GAPDH	GTGTTCCTACCCCCAATGTGT	ATTGTCATACCAGGAAATGAGCTT
Scd1	TCCTCCTTGGATTGTGTAGAAACTT	AATGTCAGAAGAAATCAGGTGGGTA
PPARγ	GAAAGACAACGGACAAATCACCAT	CGGCTTCTACGGATCGAAACTG
Adrb3	CCTTGGGCGAAACTGGTTG	GTTGGTGACAGCTAGGTAGCG
Lipe	TGGCACACCATTTTGACCTG	TTGCGGTTAGAAGCCACATAG
Pnpla2	GGATGGCGGCATTTCAGACA	CAAAGGGTTGGGTTGGTTCAG
Cpt2	CAAAAGACTCATCCGCTTTGTTC	CATCACGACTGGGTTTGGGTA
Acox1	TAACTTCCTCACTCGAAGCCA	AGTTCCATGACCCATCTCTGTC
Ppargc1α	TATGGAGTGACATAGAGTGTGCT	CCACTTCAATCCACCCAGAAAG
PPARα	TACTGCCGTTTTCACAAGTGC	AGGTCGTGTTCACAGGTAAGA
Fabp5	GGAAGGAGAGCACGATAACAAGA	GGTGGCATTGTTCATGACACA
Occludin	GTGGTTTGACACTGACTTCCC	CTCCTCTCGGTGACAGAGTCT
ZO-1	TTTTTGACAGGGGGAGTGG	TGCTGCAGAGGTCAAAGTTCAAG
Muc5	GTGGTTTGACACTGACTTCCC	CTCCTCTCGGTGACAGAGTCT
CD14	GAGTTGTGACTGGCCCAGTCAGC	GCAAAAGCCAGAGTTCCTGAC
TLR2	AAGATGCGCTTCCTGAATTTG	TCCAGCGTCTGAGGAATGC
TLR4	TGTTCTTCTCCTGCCTGACA	CATCAGGGACTTTGCTGAGTT
NLRC4	CGGCCTGCAACCTCTTTCTT	TGGGCCAAAACATTCAGGTCT
MCP-1	ATCCCAATGAGTAGGCTGGAGAGC	CAGAAGTGCTTGAGGTGGTTGTG
